# Report of Common Aeroallergens among Allergic Patients in Northeastern Iran

**Published:** 2017-03

**Authors:** Yaghoub Mahboubi Oskouei, Reza Farid Hosseini, Hamid Ahanchian, Lida Jarahi, Nazila Ariaee, Farahzad Jabbari Azad

**Affiliations:** 1 *Allergy Research Center, Mashhad University of Medical Sciences, Mashhad, Iran.*; 2 *Inflamation and Inflammatory Disease Research Center, Mashhad University of Medical Sciences, Mashhad, Iran.*; 3 *Addiction Research Center, Mashhad University of Medical Sciences, Mashhad, Iran.*

**Keywords:** Allergens, Allergic feature, Pollen, Seasonal Allergy

## Abstract

**Introduction:**

The prevalence of atopic diseases has increased in recent decades dramatically. The most common aeroallergens in Northeastern Iran have not been fully defined. Determining the most common aeroallergens in allergic patients based on the skin prick test (SPT) was aimed in this investigation.

**Materials and Methods:**

This cross-sectional study enrolled 1,006 allergic patients (aged 1–86 years) from October 2010 to February 2014 referred to the Allergy clinics of Mashhad University of Medical Science. After completing a checklist including demographic information, the SPT was performed according to the patients’ history of aeroallergen sensitivity.

**Results:**

Patients with symptoms of asthma, allergic rhinitis, atopic dermatitis, and urticaria were enrolled. Ninety seven percent of patients had a positive skin test to at least one aeroallergen. The most prevalent allergens were Russian thistle (*Salsola kali*) (50.2%), ash (*Fraxinus excelsior*) (36.7%), grass mix (29.1%), tree mix (21.6%), and pigweed mix (19.5%). Common allergens in patients with different symptoms of allergic disorders were as follows: asthma (Russian thistle, grass mix, ash, tree mix, and *Dermatophagoides pteronyssinus*); allergic rhinitis (Russian thistle, ash, grass mix, tree mix, and pigweed mix); urticaria (Russian thistle, ash, grass mix, pigweed mix, and tree mix) and atopic dermatitis (Russian thistle, grass mix, ash, tree mix, and pigweed mix). In the spring, the most prevalent allergens were Russian thistle, ash, grass mix, tree mix, and pigweed mix. In the summer, Russian thistle, ash, grass mix, tree mix, and pigweed mix accounted for the most prevalent allergens. During the autumn, Russian thistle, ash, grass mix, pigweed mix and lamb’s quarter were the most common aeroallergens, while in the winter, Russian thistle, ash, grass mix, pigweed mix, and tree mix were shown to be the most common aeroallergens.

**Conclusion:**

Determination of the most common aeroallergens in this area is unavoidable in the diagnosis and management of allergic disorders. Understanding the prevalence of the most common aeroallergens such as Russian thistle in 50.2% of people or other common aeroallergens can help patients and specialists to more easily identify suspected allergens, reduce costs, and support immunotherapy of allergic patients in this area. Moreover, it is helpful in avoiding pollens or cross-reactions.

## Introduction

Classically, allergy has been defined as the result of an immune reaction to antigens known as allergens. The genetically mediated specific immunoglobulin E (IgE) produced after exposure to allergens is called atopy, and is clinically defined as allergic sensitization to at least one allergen. Atopy is fundamental to the pathogenesis of allergic disorders, which manifest as any combination of conjunctivitis, food intolerance, asthma, rhinitis, and eczema (i.e., atopic dermatitis) (1). Individuals are exposed to a range of foreign substances in both domestic and nondomestic settings, and the term allergen is used to describe any substance capable of stimulating the production of IgE (a property known as allergenicity) in a genetically disposed individual (2).

Aeroallergens represent the most common cause of allergic disease and are derived from pollens, fungal spores, insect and mite feces, animal dander, and dust (2). Allergy skin testing using the prick-puncture method is considered to provide one of the best combinations of sensitivity and specificity (3). The purpose of this investigation was to define the prevalent aeroallergens in allergic patients based on the SPT in Mashhad, Northeast of Iran. Identifying the most common local aeroallergens will reduce costs and help create useful immunotherapies.

## Materials and Methods

*Demography: *This was a cross-sectional study among 1,006 patients with symptoms of allergic disorders who were referred to the allergy clinics of Mashhad University of Medical Science from October 2010 to February 2014. Patients without any apparent symptoms were excluded. All patients were from Khorasan, a vast area located in the Northeast of Iran with a cold semi-arid to hot-arid climate with a latitude of 36º N 59º E.

A demographic checklist including information such as age, gender, and patient history was completed, and on this basis, the exact date of the allergy was deduced in order to determine the seasonal distribution of symptomology (four groups). The two most commonplace methods for determine allergens, are the skin prick test (SPT), and the radio allegro sorbent test (RAST) in vitro and in vivo methods, respectively. As the SPT method is less time consuming and more cost effective, this was selected for our study.

*SPT: *The SPT was performed according to the patients’ history of aeroallergen hyper-sensitivity, with applying the standard allergen extract panel (GREER, USA) and compared with histamine chloride and normal saline, respectively, as positive and negative controls. Aeroallergen extracts for SPT included outdoor extracts including Russian thistle (Salsola kali) (G59), ash (Fraxinus excelsior) (P30), grass mix (TP27), tree mix (PO714), pigweed mix (P5) and lamb’s quarter (G43) and indoor extracts such as Dermatophagoides farina (D.farina) (B64), Dermatophagoides pteronyssinus (D. pteronyssinus) (B70), cockroach (BO12), Alternaria alternate (M1), Aspergillus mix (M04), penicillium (M05), *cladosporium (M13), feather (E01),and cat fur(TE3).* Choosing aeroallergens were based on Flora Iranica (4). In the SPT, a small drop of each allergen is placed on the volar surface of the forearm. A needle (25 gage) must touch each drop and penetrate into the epidermal surface at a low angle. The tip of the needle must then be gently lifted up to raise the epidermis without any bleeding, then allergen extracts should be dropped in the pricked area separately, then after about 15–20 minutes the solution is wiped away with a cotton tissue. The SPT shows a reaction which reaches a peak for allergens. The largest and smallest diameters of each reaction were measured; the average is usually reported for the result. A wheal diameter >3 mm and a flare diameter >10 mm are considered positive results (5).

Statistics: Statistical analyses were performed applying SPSS for Windows (version 22, New York, USA) as well as descriptive statistics and the Chi-square test. P<0.05 was considered statistically significant.

## Results

A total of 1,006 subjects, consisting of 56.8% female and 43.2% male patients aged between 1 and 86 years (mean, 24 years), with asthma symptoms(n=163;16.2%),allergic rhinitis(n=699; 69.5%), atopic dermatitis(n=40;4%),and urticaria (n=115;11.4%) were studied. Ninety-seven subjects (9.6%) had no definitive diagnosis and were, therefore, treated as missing values. In total, 97% of patients had a positive skin test to at least one allergen. The most prevalent allergen among patients was Russian thistle (N=505, 50.2%).The prevalence of common aeroaller- gens based on SPT can be seen in Table 1.

**Table 1:** Prevalence of common aeroallergens based on skin prick test reactivity

**Aeroallergens**	**Prevalence%(N)**
Russian thistle	50.2(505)
Ash	36.7(369)
Grass mix	29.1(293)
Tree mix	21.6(217)
Pigweed mix	19.6(196)
Lamb’s quarter	12.5(126)
Cockroach	7.5(75)
D. farina	7.1(71)
Alternaria	5.3(53)
D. pteronyssinus	5.2(52)
Aspergillus	2.4(24)
Cat fure	1.6(16)
Feather	1.4(14)
Penicillum	0.5(5)
Cladosporium	0.2(2)

Patients were divided into four groups: 0–6 years (12.7%), 6–18 years (28%), 18–50 years (53.9%), and >50 years (5.4%). 

Table 2 shows the prevalence of sensitivity to different allergens. As it can be clearly seen, weeds were most allergenic group affecting 51.9% (522) subjects, followed by trees with 42.2% (422) and grasses with 29.1% (293). Mites, cockroaches, and molds were the lowest groups, affecting 8.5% (86), 7.5% (75), and 6.8% (68) subjects, respectively. 

According to Table 2, the prevalence of sensitivity to allergens in the different seasons can be established.

**Table 2:** Prevalence of sensitivity to allergens in different seasons

**Allergen**	**Spring %(N)**	**Summer%(N)**	**Autumn%(N)**	**Winter %(N)**	**Total**
Russian thistle	14.2(70)	47.2(232)	23(113)	15.7(77)	%100
Ash	15.5(56)	46.1(167)	22.7(82)	15.7(57)	
Grass mix	18.2(52)	42.8(122)	22.1(63)	16.8(48)	
Tree mix	21.7(45)	49.8(103)	15.5(32)	13(27)	
Lamb’s quarter	24.6(31)	34.1(43)	31(39)	10.3(13)	
Cockroach	9.6(7)	41.1(30)	35.6(26)	13.7(10)	
D. farina	16.2(11)	39.7(27)	29.4(20)	14.7(10)	
D. pteronyssinus	25.5(12)	44.7(21)	21.3(10)	8.5(4)	
Pigweedmix	16.8(33)	37.8(74)	31.6(62)	13.8(27)	
Alternaria	18.9(10)	37.7(20)	24.5(13)	18.9(10)	
Aspergillus	13(3)	69.6(16)	13(3)	4.3(1)	
Cat fure	50(8)	37.5(6)	6.2(1)	6.2(1)	
feather	25(3)	50(6)	16.7(2)	8.3(1)	
Penicillum	0(0)	20(1)	60(3)	20(1)	
Cladosporium	0(0)	50(1)	50(1)	0(0)	
					

In the spring, the most prevalent allergens were Russian thistle (19.33%), ash (15.46%), grass mix (14.36%), tree mix (12.43%), and pigweed mix (9.11%). In the summer, Russian thistle (25.46%), ash (18.33%), grass mix (13.39%), tree mix (11.30%), and pigweed mix (8.12%) were the most prevalent allergens. During the autumn, Russian thistle (23.44%), ash (17.01%), grass mix (13.07%), pigweed mix (12.86%), and lamb’s quarter (8.09%) were the most common aeroallergen, while in the winter, Russian thistle (25.08%), ash (18.56%), grass mix (15.63%), pigweed mix (8.79%), and tree mix (8.79%) were the most common aeroallergens.Prevalence of sensitivity to allergens according to clinical symptoms is shown in Table 3. Russian thistle, ash, grass mix, tree mix, and pigweed mix were the most common allergens in patients with allergic rhinitis. Russian thistle, grass mix, and ash were the three most common allergens in patients with asthma, as in the previous group. They were also the most common allergens in patients with urticaria atopic dermatitis.

The most prevalent aeroallergens among the different age groups were as follows <6 years: Russian thistle (23%), tree mix (17%), grass mix (15%); 6–18 years: Russian thistle (19%), ash (16%), grass mix (14%); 18–50 years: Russian thistle (21%), ash (16%), grass mix (13%). In a group of patients who were aged above 50 years, Russian thistle (18%), ash (15%), and pigweed mix (15%) were the most common aeroallergens.

**Table 3:** Prevalence of sensitivity to allergens regarding clinical symptoms

**Clinical Symptom**	**Allergic Rhinitis**	**Asthma**	**Urticaria**	**Atopic Dermatitis**	**Total**
Russian thistle%(N)	55.3(382)	64(103)	35.1(39)	44.7(17)	%100
Ash%(N)	61.1(287)	48(73)	37.5(27)	44.4(12)	
Grass mix%(N)	47(231)	45.6(73)	25.3(20)	40(12)	
Tree mix%(N)	59.4(187)	55.1(70)	34.7(17)	42.1(8)	
Lamb’s quarter%(N)	75(75)	8(8)	16(16)	4(4)	
Pigweed mix%(N)	80(132)	6.1(10)	12.1(20)	3(5)	
Cockroach%(N)	17.1(57)	17.8(19)	21.1(8)	9.1(1)	
D. farina%(N)	28.2(67)	19.5(23)	21.4(6)	7.7(1)	
D. pteronyssinus %(N)	28.6(52)	24(24)	44.4(4)	16.7(2)	
Alternaria%(N)	11(40)	11.2(14)	7.3(3)	11.8(2)	
Aspergillus%(N)	7.6(22)	8.6(9)	5(1)	0(0)	
Cladosporium%(N)	100(2)	0(0)	0(0)	0(0)	
Penicillum%(N)	60(3)	20(1)	20(1)	0(0)	
Feather%(N)	5.9(14)	7.2(7)	0(0)	0(0)	
Cat fure%(N)	7.6(15)	8.3(6)	0(0)	0(0)	

## Discussion

In this study, 97.1% of a total of 1,006 subjects showed a positive SPT to at least one allergen, of whom 69.5% and 16.2% had allergic rhinitis and asthma, respectively. Weed pollens were the most common allergens in allergic rhinitis and asthma patients, and this is in accordance with the results reported by Kashef et al. in 2003 in Shiraz (Iran) and in contrast with the investigation conducted in Ahvaz (Iran) (6,7), which reported that dust mites were the most common aeroallergen.In all age groups, Russian thistle was the most common aeroallergen. Pigweed was more common in higher age groups (18–50 and >50 years) and in cold seasons (autumn and winter) in this study. Statistically, there was a significant relationship between allergic rhinitis and Russian thistle, ash, grass mix, tree mix, pigweed mix, lamb’s quarter and D. farina or asthma and Russian thistle, ash, pigweed mix, and penicillium. However, cross-reactions should be considered, particularly for Russian thistle which was characterized in Iran and which has a cross-eactivity with other weeds that has previously been demonstrated (8).

Skin sensitivity to at least one aeroallergen in our study occurred in 97.5% of patients, which is similar to the values of 85.6% reported by Assarehzadegan et al. in Ahvaz and 85% by Min et al. in Hawaii (9,10). Skin reaction to Russian thistle was most common among the aeroallergens, and was closely similar to results reported by Assarehzadegan et al. (9) and Fereidoni et al. (11). Sensitization to weed pollens (Russian thistle) is higher in all seasons of the year in Mashad (Iran), in contrast with Tehran (Iran) Gwangju in Korea, and in Ahwaz (Iran) (9,12,13).

In allergic rhinitis patients, weeds (Russian thistle) were the most common aeroallergens based on the SPT, consistent with results reported by Hosseini et al. and Fereidoni et al. (11,12). Allergy to Russian thistle was reported as one of the most common causes of allergic rhinitis in neighboring countries including Jordan and Kuwait (14,15). On the other hand, an investigation in Karachi (Pakistan) to the east of Iran showed somewhat different pollen counts and distribution. It should be considered that pollen counts cannot exactly represent prevalent aeroallergens, the most allergenic pollen that can affect patients being revealed in SPTs (16-17).

In our asthmatic patients, positive SPTs to aeroallergens were common due to weeds (Russian thistle) and grasses (grass mix), which is in contrast to the results reported by Hosseini et al. (12). On the other hand, other data such as the correlation of aeroallergen hypersensitivity and prevalence of urticaria or dermatitis atopic only can be reported. Our analyzing output presuming the hypothetical correlation of atopic diseases and aeroallergen hypersensitivity.

Except feather allergen, which was higher in females, there was no statistical relationship between sensitivity to aeroallergens and gender, which is comparable with the results of Kashef et al. in Shiraz and Assarehzadegan et al. in Ahvaz (6,9).

In this study, prevalence rate of D. farina and D. pteronyssinus based on SPT in all patients were 7.1% and 5.2%, respectively. These results are comparable with a study in Yazd (Iran), which reported rates of 8.4% and 7.4%, respectively (18), but is in contrast with a similar study in Mazandaran (Iran) (19). This difference may be due to the humidity ratio.

Sensitization to molds based on a SPT in our study was 6.8%. This finding is consistent with a reported 8.3% rate of mold allergy in Shiraz (6). Skin sensitivity to Alternaria in asthmatic and allergic rhinitis patients in our study was 11.2% and 11%, respectively, while in the reports of Hosseini et al. (12), the rate in allergic rhinitis patients was 32.8%.

Cockroach reported 7.5% positive SPT in all patients, which differs from a similar study in Mazandaran (Iran) and in India (19,20), possibly due to climate change (21,22). Sensitivity to cat fur allergen was found in 7.6% and 8.3% of our allergic rhinitis and asthmatic patients, respectively. Studies in Spain have reported prevalence rates of 15.5% in allergic rhinitis patients, but studies in Baltimore and Saudi Arabia have reported much higher frequencies (23-25).

This may be assigned to genetic factors or a lower exposure to cats in the Iranian population.

The rate of positive SPT to a feather mix in our study in allergic rhinitis patients was 5.9%, which is similar to studies conducted in Tehran and South Africa (12,26), which reported prevalence rates of 8.4% and 10% for sensitivity to feather allergens, respectively.

## Conclusion

Since allergens vary in different geographical areas, determination of the most common allergens and the relationship between SPT results and allergic diseases in each area is important for the diagnosis and management of allergic disorders. In this study, Russian thistle, ash, grass mix, tree mix, and pigweed mix comprised the most common aeroallergens.
